# Interleukin-1**α** Induction in Human Keratinocytes (HaCaT): An *In Vitro* Model for Chemoprevention in Skin

**DOI:** 10.1155/2012/393681

**Published:** 2012-06-25

**Authors:** T. Magcwebeba, S. Riedel, S. Swanevelder, P. Bouic, P. Swart, W. Gelderblom

**Affiliations:** ^1^Programme on Mycotoxins and Experimental Carcinogenesis (PROMEC) Unit, Medical Research Council, P.O. Box 19070, Tygerberg 7505, South Africa; ^2^Department of Biochemistry, Stellenbosch University, Private Bag X1, Stellenbosch 7602, South Africa; ^3^Biostatistic Unit, Medical Research Council, P.O. Box 19070, Tygerberg 7505, South Africa; ^4^Synexa Life Sciences, P.O. Box 1573, Bellville 7535, South Africa

## Abstract

Long-term exposure to UV irradiation and toxic chemicals is associated with chronic inflammation that contributes to skin cancer development with interleukin-1 alpha (IL-1*α*), constitutively produced by keratinocytes, playing a pivotal role in skin inflammation. The aim of this study was to investigate the modulation of IL-1*α* production in the HaCaT keratinocyte cell line. Phorbol 12-myristate 13-acetate failed to induce IL-1*α* in HaCaT cells, and this might be associated with the specific deficiency known to affect downstream signalling of the MEK/ERK pathway in these cells. The calcium ionophore, ionomycin, slightly enhanced the production of intracellular (icIL-1*α*), but this resulted in a necrotic release at higher concentrations. UV-B exposure significantly increased the production of icIL-1*α* in a dose-dependent manner with a maximal induction exhibited at 24 h with minimal necrotic and apoptotic effects. Validation of the HaCaT cell model indicated that the nonsteroidal anti-inflammatory drug (NSAID), ibuprofen, and the glucocorticoid, dexamethasone, inhibited icIL-1*α* production, and this was associated with a slight inhibition of cell viability. The UV-B-induced keratinocyte cell model provides an *in vitro* system that could, apart from phorbol ester-like compounds, be utilised as a screening assay in identifying skin irritants and/or therapeutic topical agents via the modulation of IL-1*α* production.

## 1. Introduction

Inflammation is a physiological response that protects the body against various insults, such as physical injury, pathogens, exposure to toxic chemicals, and UV irradiation. An acute inflammatory response has a therapeutic consequence when manifested over a short period of time; however, prolonged inflammation can lead to cancer development [1–3]. Various inflammatory mediators are considered to be key role players in the development of an acute and chronic response with IL-1 and TNF-*α* being the primary cytokines propagating this process [4–6]. IL-1 and TNF-*α* initiate a signalling cascade that induces the gene expression and production of secondary mediators, which include cytokines and chemokines, growth factors, adhesion molecules, cyclooxygenase type 2 (COX-2), inducible nitric oxide synthase (iNOs), and other proinflammatory factors. This facilitates the chemoattraction of reactive oxygen species (ROS)-producing immune cells which aid in the repair of damaged tissue [7, 8]. Studies have linked the role of IL-1 and TNF-*α* to the different stages of tumorigenesis with the ROS produced by immune cells mainly functioning as chemical effectors in malignant transformation [9, 10]. The chronic activation of the other secondary inflammatory mediators functions in promoting the proliferation and survival of malignant cells and also contributes to their progression as invasive tumor cells. The link between chronic inflammation and carcinogenesis indicates that opportunities exist where modulation of the primary cytokine production can be used in the development of new anti-inflammatory drugs that can be utilised as possible chemoprevention agents [9, 11].

Keratinocytes, the main epidermal cell, function as a major contributor to cytokine production [12]. Of the primary cytokines, IL-1*α* predominates, as it is constitutively synthesised as a biologically active precursor protein (proIL-1*α*) while IL-1*β* exists in an inactive precursor form [13]. In contrast, TNF-*α*, which is inducible upon exposure to stimuli, occurs at very low levels in cultured keratinocytes, and seems to play a lesser role regarding inflammation *in vivo* when compared to IL-1*α* [14, 15]. IL-1*α* remains cell-associated in keratinocytes and its biological activity, which involves wound healing and leukocyte recruitment, is regulated through its cell-expressed surface receptors. Type 1 receptor is responsible for transducing pro-inflammatory signalling while type 2 antagonises these effects and is often regarded as a “decoy” receptor [16–19]. IL-1*α* production in keratinocytes is increased by various stimuli which include phorbol esters, UV-B irradiation, and ionomycin [20–23]. Since preformed IL-1*α* is only passively released during cell injury [19], various studies have utilised this cytokine in inflammatory and carcinogenesis models as a means to investigate the molecular mechanisms involved in the progression of inflammatory skin disease and cancer promotion [21, 24–26]. The modulation of IL-1*α* has also been identified as a useful screening tool for cell irritants, and there is growing interest on the use of this cytokine in the development of anti-inflammatory drugs that can also function as chemopreventative agents [27–31].

Although the use of IL-1*α* in *in vivo* models is more physiologically relevant to humans, ethical issues and the laborious procedures involved in the use of animals have seen cultured keratinocytes become the prototype model for skin toxicity and chemoprevention studies [32–35]. In this regard, the use of transformed keratinocytes, particularly the immortalised HaCaT cell line, has been considered as a valid and easy to operate substitute for primary cultures [36]. The HaCaT cell line has been widely used in various studies related to irritancy and drug development in skin [28, 29, 36, 37]. However, recent studies have cautioned against their appropriateness in pharmacological and/or toxicity screening assays, especially when considering phorbol ester-like compound. It has been suggested that a detailed characterization of these cells should be conducted before their application as an *in vitro* model [38, 39].

The current study sought the suitability of different stimulants, including PMA, ionomycin, a calcium ionophore, and UV-B, on IL-1*α* induction utilising the immortalised transformed keratinocytic HaCaT cell line. The induction of IL-1*α* was verified by the use of known anti-inflammatory drugs in order to validate the *in vitro* cell model to be utilised as a screening assay in identifying skin irritants and/or therapeutic topical agents.

## 2. Materials and Methods

### 2.1. Chemicals and Reagents

Phorbol 12-myristate 13-acetate (PMA), dexamethasone, ibuprofen, dimethyl sulfoxide (DMSO), albumin bovine serum (BSA) (Sigma-Aldrich, USA). Fetal bovine serum (FBS) (Invitrogen, USA). RPMI-1640, Dulbeco's phosphate buffered saline (DPBS), L-glutamine, heat-inactivated fetal bovine serum, trypsin-versene, Hank's buffered salt solution (HBSS) (Lonza, Belgium). Human recombinant IL-1*α* ELISA kit (R&D systems, USA), CytoTox 96 nonradioactive cytotoxicity assay, CellTiter-Glo luminescent cell viability, Caspase-3/7 assay (Promega, USA). Triton-x100 (BDH chemical Ltd, Poole England), Tween-20 (ICN Biomedicals Inc, USA) and, ionomycin calcium salt (Synexa, South Africa).

### 2.2. Keratinocyte Cell Culture

Spontaneously immortalised keratinocytes (HaCaT) were a gift from the Department of Human Biology at the University of Cape Town, South Africa. The cells were cultured in RPMI-1640 supplemented with heat-inactivated fetal bovine serum (10%), L-glutamine (2 mM) in a humidified atmosphere of 5% CO_2_/95% air at 37°C. Cells were passaged every 3 days at a 1 : 3 split ratio.

### 2.3. Effect of PMA on the Induction IL-1*α*

#### 2.3.1. Determination of Optimal Cell Density and Incubation Time

HaCaT cells were seeded in RPMI-1640 media containing 10% FBS (100 *μ*L) at a density of 15 × 10^4^ in a 96-well microtiter plate (Corning Costar, USA) and incubated at 37°C for 24 h to reach 70–80% confluency. Fresh RPMI-1640 medium containing 0.5% FBS media containing different PMA concentrations (0.31–100 ng/mL) was used. PMA was dissolved in DMSO giving a final concentration of <0.1%, and plates were incubated for 6, 12, and 24 h, the supernatants decanted, and the cells were washed with PBS (3x). Cells were lysed with a combination of 0.5% triton in PBS and one cycle of freeze (−80°C) thawing. Cell lysates were stored at −20°C for intracellular IL-1*α* determination.

#### 2.3.2. Extracellular (exIL-1*α*) and Intracellular IL-1*α* (icIL-*α*) Induction by PMA and Ionomycin

Cells were seeded at a higher density (30 × 10^4^) to intensify the IL-1*α* signal. Cells were treated either with higher concentration of PMA (6.25-100 ng/mL) or with ionomycin (0.2–10 *μ*g/mL) and incubated for 6 h. Following incubation, supernatants were collected and stored at −20°C for exIL-1*α* determination. The cells were washed with PBS (3x) and lysed with 0.5% triton combined with one freeze-thawing cycle. Cell lysates were resuspended and kept at −20°C for the determination of icIL-1*α* production.

#### 2.3.3. IL-1*α* Determination

Both icIL-1*α* and exIL-1*α* in cell lysates and supernatants, respectively, were determined with an IL-1*α* ELISA kit according to the manufacturer's instruction. Standards were prepared and assayed in duplicates while five replicates were prepared for each sample. Absorbance was measured at 450 nm with a Dynex plate reader (Dynex technologies, USA), and data were analysed using a standard curve generated from GraphPad prism (version 5 for Windows) (GraphPad software Inc, La Jolla, CA, USA). Values were expressed as pg/mL of the supernatant or cell lysate.

#### 2.3.4. Cytotoxicity and Viability Assays (LDH Release and ATP Production)

Cytotoxicity of the tested chemicals was assessed either by monitoring LDH release or both LDH release and ATP production when determining the effect of PMA and ionomycin, respectively. For LDH release, samples were analysed utilising the colorimetric CytoTox 96 kit, and absorbance was measured at 490 nm (Dynex microplate reader). LDH release was expressed as a percentage of the total LDH released (cells were lysed by one freeze-thawing cycle) based on the following calculation:

(1) $$\text{\%}\;\text{Cytotoxicity}\;=\;\frac{{\text{Absorbance}}_{\left(\text{supernatant}\right)}}{{\text{Absorbance}}_{\left(\text{total}\;\text{LDH}\;\text{activity}\right)}}\;\times 100.$$

For determining cell viability, the CellTiter-Glo Luminescent viability kit was used to monitor ATP production in cells utilising white solid plates (Porvair Sciences, Shepperton, UK). To monitor ATP production, the luciferase reagent was added and plates rotated for 2 min and incubated at room temperature for 10 min in the dark. ATP production was monitored with the Veritas microplate luminometer (Promega, USA). The luminescent signal was measured in relative light units (RLU) and data expressed as a percentage (%) of the control cells as follows:

(2) $$\text{\%}\;\text{ATP}\;\text{production}\;=\frac{{\text{RLU}}_{\text{control}}}{{\text{RLU}}_{\text{treated}\;\text{cells}}}\;\times 100.$$

### 2.4. UV-B-Induced IL-1*α* Production, Cytotoxicity, and Apoptosis

Cells were seeded in 96-well tissue culture plate at a density of 30 × 10^4^ in media (100 *μ*L) containing 10% FBS and incubated for 24 h. After removing the cultured media, cells were exposed to different doses (20, 40, 80, 160, 240 mJ/cm^2^) of UV-B light in DPBS (100 *μ*L) without the plastic lid. The UVIlink UV crosslinker (UVitek limited, UK) was fitted with six 8 Watt UV tubes with a wavelength of 302 nm (Vilber Lourmat, France). Immediately after irradiation, treated cells were supplemented with fresh RPMI-1640 medium containing 0.5% FBS and incubated for different time periods (6, 12, and 24 h).

Cytotoxicity, apoptosis, and IL-1*α* were determined after 6, 12, and 24 h. Cytotoxicity was monitored by determining LDH release as described above. For apoptosis determination, cells were lysed with a cell lysis buffer (20 *μ*L) in combination with one freeze-thaw cycle. Cell lysates were transferred (25 *μ*L) into a white solid plate and incubated with the caspase 3/7 reagent (25 *μ*L) for 1 h in the dark at room temperature. Following incubation, plates were analysed in a Veritas microplate luminometer, and caspase-3 activity was calculated as a fold increase compared to the control.

### 2.5. Inflammatory Model Validation Utilising Anti-Inflammatory Drugs

Cells were seeded at a density of 30 × 10^4^ incubated for 24 h in RPMI-1640 medium containing 10% FBS. Cells were first exposed to UV-B light (80 mj/cm^2^) in DPBS (100 *μ*L) as described above and then incubated with varying dexamethasone and ibuprofen concentrations (0.31 to 1.25 mM) for 24 h in RPMI-1640 media containing 0.5% FBS. After the removal of the supernatants, cells were washed with DPBS and lysed with 0.5% triton combined with one cycle of freeze-thawing. Cell lysates prepared in different plates were analysed for icIL-1*α*, ATP production and LDH release as described above.

### 2.6. Statistical Analysis

All parameters were tested for normality using the Kolmogorov-Smirnof test. The homogeneity of group variances for all parametric parameters was tested using Levene's Test. Group differences for these parametric parameters were then tested using one-way ANOVA (GLM in SAS) and post hoc Tukey tests, which are post hoc multiple pairwise comparisons between the means of all the different groups. For those parameters with two or more fixed effects, interaction terms were also investigated to see if they improved model fit indicating that the interaction of main effects influences the outcome. Least squares means (LS means) were used to estimate group differences and included 95% confidence intervals for the effects and differences. The Tukey-Kramer adjustment was made automatically if the data were unbalanced.

For nonparametric parameters, significant group differences were investigated using the Kruskal-Wallis test, as well as the post hoc Tukey-type test to ascertain which groups differed from which. For parametric comparisons when only two groups were involved, *t*-tests were used.

Statistical analyses were performed with SAS v9.2, and statistical significance was considered at 5% (*P* < 0.05).

## 3. Results

### 3.1. IL-1*α* Induction by PMA

PMA is known to increase the *in vitro* production of intracellular IL-1*α* in keratinocytes at noncytotoxic concentrations [21], however, recent studies have reported PMA-specific defects in HaCaT cells, but the effect has not been demonstrated for IL-1*α* production [39, 40]. The current study investigated the modulating effect of PMA on IL-1*α* induction and release, as a function of varying concentrations and time of incubation. A significant reduction in icIL-1*α* production was noticed as a function of time with the lowest level recorded at 24 h (Table 1). However, PMA did not significantly increase intracellular IL-1*α* (icIL-1*α*) production at any of the concentrations and different time points. PMA also neither induced extracellular IL-1*α* (exIL-1*α*) release nor did it exhibit any cytotoxic effects (Table 2).

### 3.2. Effect of Ionomycin on IL-1*α* Production and Cytotoxicity

**Figure 1:**
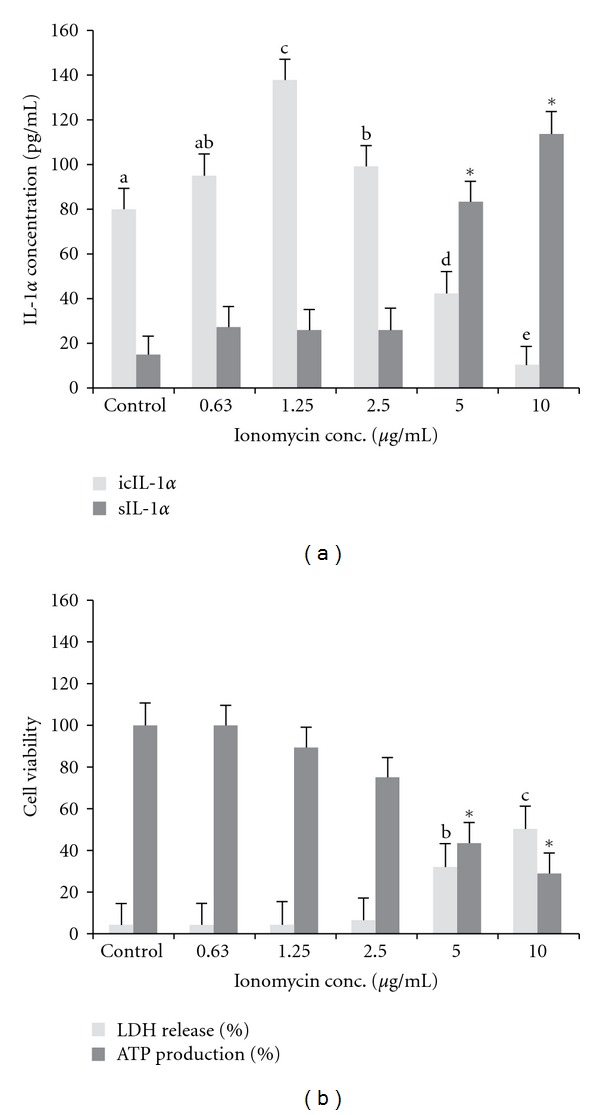
Effect of ionomycin on interleukin-1 alpha concentration in the intracellular (icIL-1*α*) and extracellular (exIL-1*α*) environment utilising HaCaT (a). Effect on cytotoxicity and cell viability monitored by the % LDH release and the % ATP production, respectively (b). Different letters or (*) on the bars indicates significant difference (*P* < 0.05) from the control. Values are the means ± SD (*n* = 4).

The ability of ionomycin to induce *de novo* synthesis and release of IL-1*α* at subcytotoxic concentrations has been demonstrated in normal keratinocytes [15]. The calcium ionophore facilitates the processing and secretion of mature IL-1*α* in monocytes and macrophages [41, 42]. The current study sought to characterise its effect on the induction and release of icIL-1*α* as a function of cell viability in HaCaT cells. Ionomycin (0.2–10 *μ*g/mL) treatment increased icIL-1*α* in a dose-dependent manner with a two-fold increase at 1.25 *μ*g/mL (Figure 1(a)). At higher ionomycin concentrations (5 and 10 *μ*g/mL), the level of exIL-1*α* was significantly increased but this was associated with a significant (*P* < 0.05) cytotoxic effect related to a reduction in cell viability (ATP production) and an increase in cytotoxicity (LDH release) (Figure 1(b)).

### 3.3. Effects of UV-B Exposure in Keratinocytes

#### 3.3.1. Dose and Time Effects of UV-B Exposure on IL-1*α* Production as a Function of Different Cell Viability Parameters

Intracellular IL-1*α* production differed significantly (*P* < 0.05) between the incubation periods, with highest level obtained after 6 h while it decreased after 12 and 24 h (Table 3). Extracellular IL-1*α* (exIL-1*α*) followed the same trend although the level was very low when compared to that of icIL-1*α*.

When considering the different UV-B treatments at 6 h, icIL-1*α* levels were unaffected at the lowest dose (20 mJ/cm^2^), while induction was effected from 40 mJ/cm^2^ onwards with the highest levels observed in cells exposed to 80 mJ/cm^2^. The icIL-1*α* levels decreased significantly at the two highest doses, but levels were still significantly higher than the untreated control. Extracellular IL-1*α* levels did not exhibit response to UV-B when compared to the unexposed cells. After 12 hr, the lowest two UV-B dosages had no significant effect on icIL-1*α* levels, while cells exposed to 80 mJ/cm^2^ effected the highest icIL-1 production. However, at the two highest UV-B doses, the induction was significantly (*P* < 0.05) reduced in a dose-dependant manner reaching levels that were similar to the untreated control cells. In general the icIL-1*α* levels significantly decreased when compared to the 6 h levels. Regarding exIL-1*α*, there was a marked increase in the levels from 40 to 240 mJ/cm^2^ which became significant (*P* < 0.05) at the two highest doses. After 24 h, cells exhibited a similar pattern to that observed at 12 h when considering the induction of icIL-1*α*. In contrast to the earlier time points, the 160 mJ/cm^2^ dose yielded similar results to 80mj/cm^2^ exposure treatment. However, at 240 mJ/cm^2^, icIL-1*α* was significantly reduced to levels that were similar to the untreated control cells. This reduction coincided with a significant (*P* < 0.05) increased in the exIL-1*α* levels similar to the 12 h time point. Of interest was the fold increase of icIL-1*α* in cells exposed to 80 mJ/cm^2^ and 160 mJ/cm^2^ which was markedly higher (3-fold) at 24 h when compared to the 6 and 12 h time points (2-fold).

When considering the % LDH release as a function of cytotoxicity, no effect was noticed after 6 h. However, at 12 h, a dose-dependent effect became evident as a slight increase in LDH release was observed in the UV-B-treated cells. At 24 h, an increase in LDH release was effected at 80 mj/cm^2^ with a maximal activity observed at the two highest doses (160 mJ/cm^2^ and 240 mJ/cm^2^). Comparison of the cytotoxicity effects between the different incubation periods indicated no significant difference between 6 and 12 h while the highest doses displayed a significant increase in cytotoxicity at 24 h.

The UV-B-induced caspase-3 activity also varied when considering the different incubation periods and dose of exposure. When considering caspase-3 activity at 6 h, a 2-fold increase was observed from 80 mJ/cm^2^ and above. After 12 h, a significant fold increase (2x) was noticed from 40 mj/cm^2^ with a dose response increase of up to 15-fold obtained with the highest dose of 240 mJ/cm^2^. The icIL-1*α*-to-apoptosis ratio, considering the fold increase, was 1 : 3 at the 80 mJ/cm^2^ dose of exposure. After 24 h the apoptotic fold increase was only observed from 80 mJ/cm^2^ and above. The fold increase in apoptosis significantly (*P* < 0.05) decreased when compared to the 12 h time point with a icIL-1*α*-to-apoptosis ratio of approximately 1 : 1 similar to that obtained after 6 h.

#### 3.3.2. Anti-Inflammatory Activity (Dexamethasone and Ibuprofen)

To investigate the modulation of the UV-induced icIL-1*α* production in HaCaT cells, the inhibitory effects of known anti-inflammatory compounds were used to validate the cell model as screening tool in identifying compounds to be utilised as chemopreventive agents. Dexamethasone inhibited UV-induced icIL-1*α* production after 24 h in a dose-dependent manner while ibuprofen only exhibited the strongest inhibitory effect at the highest concentration used (Table 4). Ibuprofen (1.25 mM) exhibited the strongest inhibition when compared to dexamethasone with icIL-1*α* reduced to levels similar to the control. However, the opposite was true at the lower concentrations. The inhibition of icIL-1*α* by dexamethasone was not associated with a significant decrease in ATP production when compared to the UV-B-treated cells while the LDH release was significantly lower and comparable to the untreated control keratinocytes. However, the inhibitory effect of ibuprofen was associated with a significant decrease in ATP production when compared to the treated keratinocytes. The LDH release was significantly lower than the UV-B-treated cells and exhibited levels similar to the untreated cells.

## 4. Discussion

Cytokine production by keratinocytes has been investigated *in vitro* utilising primary and a variety of keratinocyte cell lines [12, 13, 15, 28, 43]. These studies have established that IL-1*α* is produced as a biologically active precursor protein in both mouse and human keratinocyte and that injured human keratinocytes release preformed IL-1*α* which can be used to predict the effect of external stimuli on the *in vivo* inflammatory processes and skin carcinogenesis [15, 27, 34, 43]. *In vitro* models showed that the biological activity of IL-1*α*, induced by phorbol esters and UV-B, can be modulated by various agents including synthetic and natural compounds [28, 29, 44], thus indicating that IL-1*α*, or the modulation thereof, can play a significant role in the treatment of inflammatory disease and chemoprevention in skin. However, data obtained from these *in vitro* keratinocyte models should be interpreted with care as differences exist between normal keratinocytes and transformed cell lines [38, 39]. Of particular importance is the HaCaT cell line which is widely used as a screening tool in irritancy and chemoprevention studies. This immortalised spontaneously transformed cell line has been reported to have dysregulated molecular mechanisms related to carcinogenesis/chemoprevention [38, 39]. As a result it has been suggested that the characterization of HaCaT cells and other transformed cell lines should be critically evaluated before application as an in* vitro* model in skin toxicity and chemoprevention studies. Thus the present study aimed to characterize the induction of IL-1*α* and the resulting effect on cell integrity following exposure to PMA, ionomycin, and UV-B irradiation.

Although the skin cancer promoter PMA is known to induce the gene expression and IL-1*α* production in normal keratinocytes [20, 21], HaCaT cells have been shown to have a PMA-specific defect that is associated with an impaired mitogen-activated protein kinases (MAPKs) down-stream to the MEK/ERK signalling pathway [39, 42]. However, PMA signalling involved in the induction of IL-*α* gene expression in mouse keratinocytes has been suggested to occur via protein kinase C and not MAPK [20]. In the present study PMA did not induce IL-1*α* production, suggesting that dysregulated PMA signalling in HaCaT cells is not only specific to MAPK but also involves other signalling pathways. Therefore, HaCaT cells may not be a useful model to investigate the effect of phorbol ester-like chemicals in skin toxicity and/or on cytokines induction. However, it is known that HaCaT cells differentiate normally [45] and are known to provide the microenvironment relevant to skin sensitization, for example, by responding to epidermal growth factor, transforming growth factor (TGF)-*β*1, TGF-*α*, IL-6, interferon gamma, and TNF-*α*, to stimulate the production of IL-1*α* [36, 46]. The HaCaT cell model was also found to be a suitable *in vitro* model to investigate phase I and phase II epidermal metabolism of chemicals and drugs [36].

The calcium ionophore, ionomycin, has been reported to initiate the processing of precursor IL-1*α* into the active cytokine in many cell types via the action of calpain, a calcium-dependent membrane-associated protease [41, 42]. Ionomycin augmented the proteolytic processing of preformed IL-1*α* and release of mature IL-1*α* in lipopolysaccharide- (LPS-) activated cell line that constituively produces human IL-1*α* [41]. In keratinocytes, however, constitutive icIL-1*α* is rarely secreted in the full-length precursor form, but subcytotoxic concentrations of ionomycin have been shown to increase icIL-1*α* levels associated with the *de novo* synthesis [15]. On the other hand, the presence of IL-1*α* at low levels in the extracellular environment implicates secretion of 17 kDa mature protein, the processing of which is facilitated by ionomycin via the calcium-dependent calpain proteases [47]. In the present study, ionomycin increased the intracellular IL-1*α* level at lower concentrations, and this was accompanied by a slight release into the supernatant; however, at higher concentrations there was a cytotoxic release. This is in agreement with other studies indicating that IL-1*α* is only released under certain severe pathological conditions associated with cell death [48]. The uncontrolled and/or necrotic release of IL-1*α* may not be appropriate for chemoprevention studies concerned with a “controlled” response as the former exacerbates inflammation. Unlike the processed mature IL-1*α*, icIL-1*α* protein is nonsecretable, but during necrosis it “leaks” out to the extracellular environment where it augments the inflammatory activity in cells through receptor signalling [49].

UV-B exposure of HaCaT cells resulted in a dose-dependent increase in the level of icIL-1*α* with the release into the external environment associated with a slight cytotoxic effect at higher-dose levels. This is in agreement with a similar study that was conducted in primary keratinocytes where IL-1*α* was reported to increase intracellular at nonlethal doses while it was progressively released at lethal doses (>100 mJ/cm^2^) due to membrane damage [50]. Optimal fold induction of icIL-1*α* was achieved after 24 h at a UV-B dose of 80 mJ/cm^2^ with minimal cytotoxicity and a low level of apoptosis when compared to the 12 h time point. Induction of apoptosis by UV-B light in HaCaT cells corresponds with another study which provided evidence that UV-induced apoptosis proceeds via the intrinsic (mitochondrial) and extrinsic pathways [51]. The extent of apoptosis will depend on the amount of DNA damage induced by UV-B, and modulation of the latter could have a primary influence on the induction of skin carcinogenesis.

IL-1*α* is suggested to exhibit a dual function, that is, intracellular related to the activation of transcriptional machinery with its effect on the progression of inflammation and its extracellular receptor-mediated signal transduction [49, 52]. In UV-B-exposed keratinocytes, it was shown that IL-1*α* not only regulates its own production but also increases the production of other proinflammatory cytokines such as TNF-*α*, IL-6, and IL-8 [53]. Modulation of the intracellular function of IL-1*α*, therefore, seems to be a useful target in some inflammatory conditions [52]. Inactivation of the biologically active IL-1*α*, via the induction of apoptosis for instance could prevent inflammation since it is retained in the chromatin fraction and not released with the cytoplasmic content [49]. This suggests that UV-induced IL-1*α* production could be a useful model to modulate inflammation thereby disrupting mechanisms related to inflammatory diseases via the modulation of apoptosis.

UV-B irradiation is also known to induce the release of natural IL-1 inhibitors, epidermal cell contra-IL-1 and the endogenous IL-1 receptor antagonist (IL-1ra) in keratinocytes [54, 55]. The release of these molecules is implicated in the regulation of immune and proliferative responses by blocking IL-1 activity. IL-1ra has been studied extensively [47], and its antagonist activity is attributed to a specific ratio between IL-1ra/IL-1*α* which can be modulated by other cytokines and various agents including UV-B irradiation [46, 56, 57]. Modulation of the IL-1ra/IL-1*α* ratio by UV-B is dependent on the dose where low doses are associated with high levels of IL-1ra while higher doses shift the balance towards IL-1*α* [57, 58]. An imbalance towards IL-1*α* is associated with inflammatory disease including skin carcinogenesis [56] as it was shown that the IL-1ra/IL-1*α* ratio differs between the different stages of carcinogenesis. The highest levels of IL-1ra were found in cells representing the early stages of skin carcinogenesis where it is implicated in antitumor activity [59]. Modulating the ratio between IL-1ra/IL-1*α* offers an alternative mechanism that can be explored and incorporated into developing chemoprevention cell models *in vitro*.

The inhibitory effect by the glucocorticoid (GR), dexamethasone, and the anti-inflammatory drug, ibuprofen, on IL-1*α* production showed that the model could also be used to test anti-inflammatory effects, apart from the induction of apoptosis. Dexamethasone is known to activate cytosolic GR receptor that antagonises activity of transcription factors such as activated protein-1 (AP-1), nuclear factor kappa beta (NF-*κ*B), and these are required for the gene expression and production of proinflammatory cytokines [60]. Ibuprofen has also been reported to downregulate the production of proinflammatory cytokine at high doses, apart from its effect on cyclooxygenase downstream [61]. As the inhibition of IL-1*α* in the current study was associated with a reduced cell metabolic activity in the absence of any cytotoxicity, the modulation of apoptotic cell death should be further investigated.

The current UV-B/HaCaT *in vitro* model provides ample opportunities to regulate the production of IL-1*α*, either by disrupting the signal transduction pathways related to its induction and/or by enhancing apoptosis thereby indirectly removing cells containing excessive levels of the primary cytokine. Except for the activity of phorbol ester-like compounds, HaCaT cells behave similar to normal skin keratinocytes, especially in their response to UV-B exposure and could therefore be used to monitor the modulation of IL-1*α* content by potential anti-inflammatory and or proapoptotic compounds. However, subsequent validation utilising different *in vivo* systems in mouse and human skin should be used to verify their modulating properties prior to developing possible therapeutic products for modulating IL-1*α* production.

## Figures and Tables

**Table 1:** Effect of different incubation times and PMA concentrations on the intracellular production of IL-1*α*.

PMA conc. (ng/mL)	Ctrl.	6.25	12.5	25	50	100
Exposure (h)	IL-1*α* concentration (pg/mL)					
6	168.85 ± 32.57^a^_A_	132.75 ± 6.07^a^_A_	203.97 ± 8.97^a^_A_	180.16 ± 12.27^a^_A_	189.38 ± 16.36^a^_A_	162.66 ± 27.76^a^_A_
12	112.59 ± 0.83^a^_B_	60.64 ± 10.49^b^_B_	71.22 ± 8.99^ab^_B_	86.85 ± 11.71^ab^_B_	75.06 ± 9.90^ab^_B_	73.73 ± 4.07^ab^_B_
24	15.69 ± 1.15^a^_C_	9.61 ± 0.48^a^_C_	10.29 ± 0.66^a^_C_	9.82 ± 2.48^a^_C_	9.15 ± 1.38^a^_C_	9.61 ± 0.37^a^_C_

Cells were seeded at a density of 15 000 cells/well. Values represent the mean ± standard deviation of four replicates. Letters in superscript (row) indicate significant different between control and PMA concentrations; letters in subscript (column) indicate significant difference between incubation periods. Values were considered significant when *P* < 0.05.

**Table 2:** Effect of PMA on the intracellular and extracellular IL-1*α* production and determination of cytotoxic effects.

PMA conc. (ng/mL)	Ctrl.	6.25	12.5	25	50	100
	IL-1*α* concentration (pg/mL)					
Intracellular	256.30 ± 26.24^a^_A_	266.71 ± 25.06^a^_A_	223.29 ± 82.14^a^_A_	200.91 ± 69.97^a^_A_	234.73 ± 31.16^a^_A_	233.17 ± 36.38^a^_A_
Extracellular	1.74 ± 0.23^a^_B_	2.60 ± 0.87^a^_B_	1.78 ± 0.696^a^_B_	2.28 ± 0.81^a^_B_	1.86 ± 0.50^a^_B_	1.93 ± 0.40^a^_B_
**% LDH release**	7.12 ± 0.93^**a**^	7.90 ± 1.65^**a**^	6.91 ± 0.68^**a**^	9.48 ± 2.67^**a**^	6.52 ± 0.65^**a**^	7.22 ± 0.69^**a**^

Cells were seeded at a density of 30 × 10^4^/well and incubated for 6 h. Values represent the mean ± standard deviation of 6 replicates. Cytotoxicity represents LDH release as a percentage of the total. Letters in superscript indicated significant differences between control and PMA concentration; letters in subscript indicated significant differences between intracellular and extracellular IL-1*α* production. *P* < 0.05 was considered significant. PMA: phorbol 12-myristate 13-acetate; LDH: lactate dehydrogenase.

**Table 3:** UV-induced IL-1*α* production, cytotoxic, and apoptotic effects.

Incubation period	Unit of measure	UV dose (mJ/cm^2^)					
		Ctrl	20	40	80	160	240
6 h	icIL-1*α* (pg/mL)	78.52 ± 8.50^a^_Aa_	65.56 ± 10.45^a^_Aa_	102.16 ± 18.78^b^_Aa_	159.66 ± 32.91^c^_Aa_	113.40 ± 28.04^b^_Aa_	110.96 ± 26.28^b^_Aa_
	**icIL-1*α* fold increase**	1.00 ± 0.11^**a**^_**a**_	0.83 ± 0.13^**a**^_**a**_	1.30 ± 0.24^**b**^_**a**_	2.03 ± 0.42^**c**^_**a**_	1.44 ± 0.36^**d**^_**a**_	1.29 ± 0.46^**b**^_**a**_
	exIL-1*α* (pg/mL)	14.57 ± 4.1^a^_B_	14.72 ± 4.37^a^_B_	17.39 ± 4.58^a^_B_	12.57 ± 2.76^a^_B_	16.21 ± 4.44^a^_B_	18.91 ± 5.57^a^_B_
	% LDH release	8.69 ± 1.64^a^_a_	8.39 ± 3.10^a^_a_	11.55 ± 2.79^a^_a_	11.12 ± 3.69^a^_a_	11.90 ± 1.58^a^_a_	11.82 ± 2.25^a^_a_
	Caspase-3 (fold increase)	1.00 ± 0.06^a^_a_	1.08 ± 0.08^a^_ba_	1.54 ± 0.34^b^_ab_	2.55 ± 0.28^c^_a_	2.96 ± 0.55^c^_a_	2.56 ± 0.12^c^_a_

12 h	icIL-1*α* (pg/mL)	54.69 ± 10.50^a^_Aa_	43.18 ± 11.67^a^_Aa_	37.81 ± 12.01^a^_Ab_	148.36 ± 38.69^b^_Aa_	104.12 ± 23.10^c^_Aa_	51.27 ± 12.49^a^_Ab_
	**icIL-1*α* fold increase**	1.00 ± 0.19^**a**^_**a**_	0.79 ± 0.21^**a**^_**a**_	0.69 ± 0.22^**a**^_**a**_	2.71 ± 0.71^**b**^_**b**_	1.90 ± 0.42^**c**^_**b**_	0.94 ± 0.23^**d**^_**a**_
	exIL-1*α* (pg/mL)	6.30 ± 1.64^a^_B_	6.97 ± 1.35^a^_B_	10.32 ± 4.72^a^_B_	9.85 ± 2.71^a^_B_	9.04 ± 1.71^b^_B_	11.14 ± 1.56^b^_B_
	% LDH release	6.59 ± 2.51^a^_a_	10.13 ± 0.81^b^_a_	11.33 ± 1.33^b^_a_	12.72 ± 2.56^b^_a_	13.11 ± 2.67^b^_a_	13.93 ± 2.48^b^_a_
	Caspase-3 (fold increase)	1.00 ± 0.05^a^_a_	1.28 ± 0.24^a^_a_	2.09 ± 0.68^b^	6.86 ± 0.64^c^_b_	12.55 ± 0.82^d^_b_	15.32 ± 1.16^d^

24 h	icIL-1*α* (pg/mL)	17.49 ± 6.65^a^_Ab_	18.45 ± 3.74^a^_Ab_	22.115.95^a^_Ac_	55.01 ± 11.61^b^_Ab_	55.76 ± 6.15^b^_Ab_	38.15 ± 9.25^a^_Ab_
	**icIL-1*α* fold increase**	1.03 ± 0.39^**a**^_**a**_	1.05 ± 0.21^**a**^_**a**_	1.26 ± 0.34^**b**^_**a**_	3.14 ± 0.66^**b**^_**c**_	3.19 ± 0.35^**b**^_**c**_	2.18 ± 0.53^**c**^_**b**_
	exIL-1*α* (pg/mL)	5.89 ± 1.89^a^_B_	6.67 ± 2.21^a^_B_	7.54 ± 1.18^a^_B_	5.29 ± 2.19^a^_B_	13.94 ± 3.08^b^_B_	14.97 ± 4.27^b^_B_
	% LDH release	8.24 ± 1.15^a^_a_	9.27 ± 1.49^a^_a_	11.05 ± 1.87^a^_a_	15.43 ± 3.84^b^_a_	25.71 ± 6.26^c^_b_	25.53 ± 4.50^c^_b_
	Caspase-3 (fold increase)	1.00 ± 0.08^a^_a_	1.42 ± 0.11^b^_a_	1.40 ± 0.12^b^_a_	3.25 ± 0.54^c^_a_	3.92 ± 0.46^c^_a_	3.73 ± 0.21^c^_b_

Cells were seeded at a density of 30 × 10^4^/well; values represent mean ± standard deviation of quadruplicates; small letters in superscript indicates difference between control and UV dose (mj/cm^2^); capital letter, in subscript indicates significant difference between intracellular IL-1*α* and extracellular IL-1*α* (pg/mL); small letter in subscript indicate significant difference between incubation periods (hours) for icIL-1*α* (pg/mL), icIL-1 fold increase, toxicity, and capsase-3; Values were considered significant if *P* < 0.05. icIL-1*α*: intracellular interleukin 1*α*; exIL-1*α*: extracellular interleukin 1*α*; LDH: lactate dehydrogenase.

**Table 4:** Effect of dexamethasone and ibuprofen on icIL-1*α* production, cell viability and cytotoxicity.

Dexamethasone conc (mM)	Ctrl	Positive Ctrl	1.25	0.63	0.31
icIL-1*α* (pg/mL)	19.40 ± 2.50^a^	76.04 ± 7.20^b^	48.90 ± 9.81^c^_a_	67.70 ± 10.40^b^_a_	67.60 ± 9.22^b^_a_
(% inhibition)	—	—	40.77 ± 5.40	15.41 ± 7.80	17.05 ± 12.02
% ATP production	100.00 ± 3.25^a^_a_	85.65 ± 6.12^b^_a_	76.39 ± 6.09^b^_a_	78.69 ± 4.63^b^_a_	86.59 ± 3.37^b^_a_
% LHD release	3.48 ± 0.83^a^_a_	8.84 ± 0.96^c^_a_	5.51 ± 0.41^b^_a_	5.49 ± 0.92^b^_a_	4.45 ± 0.71^b^_a_

Ibuprofen conc (mM)	Ctrl*	Positive Ctrl*	1.25	0.63	0.31

icIL-1*α* (pg/mL)	—	—	20.67 ± 3.69^a^_b_	86.84 ± 11.27^b^_b_	86.45 ± 12.77^b^_b_
(% inhibition)	—	—	72.89 ± 5.60	(—)	(—)
% ATP production	100.00 ± 3.80^a^_a_	90.19 ± 9.38^a^_a_	64.76 ± 5.89^b^_b_	84.75 ± 11.99^b^_a_	88.98 ± 14.01^a^_a_
% LDH release	4.65 ± 0.19^a^_a_	10.17 ± 0.84^c^_a_	5.77 ± 0.59^b^_a_	5.30 ± 0.55^ab^_a_	4.75 ± 0.72^ab^_a_

Ctrl-control represents cells that have not been exposed to UV-B light. Values presented are the mean ± standard deviations. letters in subscript indicate significant difference between treatment concentration of dexamethesone and ibuprofen; values in italics represent % inhibition of icIL-1*α* by dexamethasone and ibuprofen (—) indicates no inhibition; % inhibition calculated from the positive control. Effect of dexamethasone and ibuprofen was conducted in the same experiment therefore, similar control and pos control were used. *Pos Ctrl- Positive control represents cells exposed to 80 mj/cm^2^ of UV-B light. icIL-1*α* intracellular interleukin-1alpha.
